# Glaucomatous and Age-Related Changes in Corneal Pulsation Shape. The Ocular Dicrotism

**DOI:** 10.1371/journal.pone.0102814

**Published:** 2014-07-17

**Authors:** Monika E. Danielewska, Patrycja Krzyżanowska-Berkowska, D. Robert Iskander

**Affiliations:** 1 Institute of Biomedical Engineering and Instrumentation, Wroclaw University of Technology, Wroclaw, Poland; 2 Department of Ophthalmology, Wroclaw Medical University, Wroclaw, Poland; Bascom Palmer Eye Institute, University of Miami School of Medicine, United States of America

## Abstract

**Purpose:**

To ascertain whether the incidence of ocular dicrotic pulse (ODP) increases with age, it is more pronounced in glaucomatous than in normal eyes and whether it is related to cardiovascular activity.

**Methods:**

261 subjects aged 47 to 78 years were included in the study and classified into four groups: primary open angle glaucoma (POAG), primary angle-closure glaucoma (PACG), glaucoma suspects with glaucomatous optic disc appearance (GODA) and the controls (CG). Additionally, in each group, subjects with ODP were divided into two age subgroups around the median age. A non-contact ultrasonic method was used to measure corneal indentation pulse (CIP) synchronically with the acquisition of electrocardiography (ECG) and blood pulse signals. ODP was assessed from the acquired signals that were numerically processed in a custom written program.

**Results:**

ODP incidence was about 78%, 66%, 66% and 84% for CG, GODA, POAG, and PACG group, respectively. With advancing age, the ODP incidence increased for all subjects (Δ = 12%), the highest being for the PACG and POAG groups (Δ = 30%). GODA group did not show an age-related increase in the incidence of ODP.

**Conclusions:**

The ocular dicrotism, measured with non-contact ultrasonic method, was found to be a common phenomenon in elderly subjects. The increased ODP incidence in PACG and POAG group may correspond to either higher stiffness of glaucoma eyes, biochemical abnormalities in eye tissues, changes in ocular hemodynamics, may reflect the effect of medications or be a combination of all those factors. The results of GODA group suggest different mechanisms governing their ocular pulse that makes them less susceptible to generating ODP and having decreased predisposition to glaucoma.

## Introduction

Aging causes physiological, functional and structural changes in the human eye. It is manifested in variations in elasticity of the eye tissues (cornea and sclera) [1]−[6], vascular ocular hemodynamics [7]−[9] biometric parameters of the eye globe (e.g., smaller anterior chamber volume) [10], decreased aqueous production [11], as well as an increased latency of saccadic eye movements [12]. Majority of these factors are linked to the ocular rigidity phenomenon, which was reported to increase with age [13]. Also, it is a parameter which affects the measurement of ocular pulse characteristics [14]. The ocular pulse describes the natural changes in intraocular pressure (IOP) and ocular blood pulsation [15]−[18], which were found to be related [19], [20], follow some autoregulation processes [21], and may depend on eye-globe geometry [22]. Further, the ocular pulse was found to be highly influenced by heart activity [23]−[27].

One of the ocular pulse signals is a corneal indentation pulse (CIP), defined as temporal variations in corneal expansion [28], [29]. It should be noted, however, that it is also a superposition of longitudinal eye movement in the orbit and the actual expansion due to the net increase in the ocular blood volume [26]. Investigation of changes in the waveform of the CIP signal has indicated associations with corresponding pathological alterations in ocular blood supply [28]. Our recent results, based on a new noninvasive ultrasonic method [29], revealed that in the substantial proportion of older subjects (ranged from 44 to 72 years old) a double-peak-shaped character of the CIP signal was found. This phenomenon, termed the ocular dicrotism, was not evident in the corresponding younger group of subjects (ranged from 23 to 32 years old).

In a range of world’s populations, a strong positive correlation between age and glaucoma was observed [30]−[32]. However, it is not fully elucidated, which aspects of aging are responsible for the increased susceptibility to glaucoma.

As the world population ages, glaucoma is a prominent cause of blindness [33]. Estimated over 8 million people are blind as the result of glaucoma [34]. Number of glaucomatous and suspect subjects is still increasing. Nowadays, besides increased IOP and visual field loss, hemodynamics of the eye has become a key factor in the diagnosis of early stage glaucoma [35]. Hence, investigation of age-related relationships between changes in shape of the ocular pulse signals and cardiovascular activity signals might be a promising step to gain more information about ocular hemodynamics aspects of glaucoma progression. There is a range of techniques available for measuring the ocular pulse. They include contact and non-contact techniques such as, pneumatic tonometry [17], [36], ocular pneumoplethysmography [24], [37], ultrasonography [15], [16], scanning laser Doppler flowmetry [38], optical coherence tomography [39], spectral-domain low coherence interferometry [40] and dynamic contour tonometry [41].

In this study, we use recently proposed innovative ultrasonic method to register in a non-contact manner and continuously the ocular pulse characteristic, as the CIP signal, simultaneously with the heart activity signals (blood pulse and electrocardiogram) [29], [42]. The CIP signal parameters were shown to be highly correlated with the parameters of electrical heart activity [27].

The aim of this study was to answer the following questions:

Q1. Does the incidence of ocular dicrotic pulse (double-shaped pulse in CIP signal) in elderly subjects is a function of age?
Q2. Is the ocular dicrotism a more common phenomenon in the glaucomatous eyes than in the normal ones?
Q3. Can the parameters of the CIP signal together with the parameters of cardiovascular activity provide more insight into the phenomenon of ocular dicrotism?

## Materials and Methods

### Participants

One hundred ninety one patients aged from 47 y.o. to 78 y.o. (mean ± SD, 63.7±8.4) participated in this study. They were enrolled from the regular patients of the Glaucoma Clinic at the Department of Ophthalmology, Wroclaw Medical University. The control group (CG) was formed with 70 volunteers aged from 50 y.o. to 74 y.o. (mean ± SD, 61.2±5.3), who did not exhibit any ocular and cardiovascular abnormalities. Forty one out of 70 subjects from the control group participated in earlier experiment [29]. They were recruited from the university staff and their families.

Before the measurements, the purpose of this study and the study protocol were explained to the participants. Signed informed consent form was obtained from all of the subjects and patients. All measurements and procedures were conducted in collaboration with the Wroclaw Medical University. The study was approved by the Ethics Committee of Wroclaw Medical University (decision No KB 503/2011) and adhered to the Tenets of the Declaration of Helsinki.

All subjects from the Glaucoma Clinic underwent general medical history review and an extensive ophthalmic examination including: refraction, visual acuity measurement, central corneal thickness (CCT), slit lamp biomicroscopy, Goldmann applanation tonometry (GAT), gonioscopy and examination of the optic disc. Additionally, the optic nerve head (ONH) was imaged by SD-OCT (Spectralis, Heidelberg Engineering GmbH, Heidelberg, Germany). The inclusion criteria for patients consisted of the following: visual acuity 6/12 or better, spherical refraction up to 5 diopters, and cylinder correction within 3.0 D. The Spaeth gonioscopy grading system was used to evaluate the anterior chamber angle configuration. Subjects were excluded if they had a history of ocular surgery within 6 months before the onset of the study. Patients with intraocular disease (e.g., diabetic retinopathy, retinal vein occlusion) or neurological disorders affecting visual fields were also excluded from the study. The healthy volunteers were screened by the glaucoma specialist to ensure they conformed to the study requirements and were categorized as the control group (CG).

Finally, the screened subjects were divided into three categories, within which they were diagnosed as:

1. glaucoma suspects with glaucomatous optic disc appearance (GODA)
2. primary open angle glaucoma (POAG)
3. primary angle closure glaucoma (PACG)

Subject main group statistics are given in Table 1.

**Table 1:** Number of subjects, mean age and gender ratio of subjects with single and double-peak-shape in CIP signal (ODP) for the four considered groups.

Subject Group	CG		GODA		POAG		PACG		All	
Variables	Single CIP	Double CIP(ODP)	Single CIP	Double CIP(ODP)	Single CIP	Double CIP(ODP)	Single CIP	Double CIP(ODP)	Single CIP	Double CIP(ODP)
**Number of Subjects**	16	54	21	39	24	46	11	50	72	189
**(Male/Female)**	(6/10)	(34/20)	(14/7)	(13/26)	(4/20)	(18/28)	(4/7)	(16/34)	(28/44)	(81/108)
**Mean age: years±SD**	58.5±6.6	61.9±4.6	67.0±8.0	66.5±10.7	60.3±5.1	59.9±7.5	59.9±8.2	66.2±7.1	61.8±7.2	63.5±8.0
**(range)**	(50÷68)	(52÷74)	(55÷78)	(47÷78)	(53÷78)	(48÷78)	(50÷78)	(50÷78)	(50÷78)	(47÷78)
**Mean GAT IOP** **(mmHg±SD)**	16.3±2.7	14.0±3.0	14.7±2.6	14.8±3.1	16.4±2.7	15.3±2.5	17.8±3.5	16.0±3.8	16.2±3.0	15.0±3.2
**(range)**	(10÷21)	(7÷21)	(11÷20)	(9÷21)	(11÷21)	(11÷24)	(12÷22)	(9÷25)	(10÷22)	(7÷25)
**Mean CCT (µm±SD)**	575±44	567±36	561±33	537±34	550±37	531±47	554±15	560±29	558±35	550±40
**(range)**	(513÷640)	(515÷640)	(515÷621)	(467÷594)	(516÷631)	(424÷622)	(534÷572)	(522÷621)	(513÷640)	(424÷640)
**Mean MD±SD**	-	-	–1.0±2.0	–2.0±1.9	–2.7±4.0	–3.6±5.2	–2.4±4.0	–5.7±8.9	-	-
**(range)**	-	-	(–5.5÷1.4)	(–8.5÷–0.1)	(–13.7÷1.8)	(–16.9÷1.8)	(–9.6÷0.8)	(–26.2÷1.3)	-	-
**Mean C/D±SD**	-	-	0.39±0.10	0.44±0.12	0.44±0.16	0.46±0.15	0.41±0.16	0.44±0.23	-	-
**(range)**	-	-	(0.26÷0.51)	(0.33÷0.74)	(0.22÷0.70)	(0.18÷0.71)	(0.11÷0.53)	(0.11÷0.80)	-	-
**Systolic blood pressure**	140±18	138±20	137±20	139±18	139±19	142±18	140±18	143±19	139±19	140±20
**(range)**	(93÷191)	(89÷185)	(90÷184)	(103÷188)	(96÷176)	(103÷196)	(93÷185)	(96÷180)	(93÷191)	(89÷196)
**Diastolic blood pressure**	77±8	76±11	82±11	80±12	80±11	76±8	78±11	75±9	79±10	77±11
**(range)**	(64÷91)	(59÷101)	(58÷103)	(63÷101)	(62÷104)	(60÷89)	(58÷95)	(58÷93)	(58÷104)	(58÷101)
**Mean HR bp±SD**	75±14	75±12	73±7	73±8	73±9	78±10	80±6	70±14	75±10	74±12
**(range)**	(54÷120)	(54÷120)	(60÷90)	(60÷90)	(60÷96)	(60÷96)	(72÷90)	(42÷96)	(54÷120)	(54÷120)

CIP – Corneal Indentation Pulse; ODP – ocular dicrotic pulse; CG – control group; GODA – glaucoma suspects with glaucomatous optic disc appearance; POAG – primary open angle glaucoma; PACG – primary angle closure glaucoma; GAT – Goldmann applanation tonometry.

Controls consisted of those having no history of ocular disease, no family history of glaucoma, IOP measurements of 21 mmHg or less, normal optic disc and normal visual field.

GODA was defined as the presence of any sign of glaucomatous optic nerve damage (concentric enlargement of the optic cup, neuroretinal rim thinning or notching) with normal visual field and normal IOP.

POAG was defined as the presence of glaucomatous optic nerve damage (i.e., concentric enlargement of the optic disc, presence of focal thinning, or notching) with associated visual field defects and high or normal IOP in the presence of an open angle.

PACG was defined as the presence of glaucomatous optic nerve damage corresponding to visual field defects and anatomically narrow angle with or without synechia.

All subjects exhibited subnormal levels of IOP (mean ± SD, 15.6±3.5 mmHg), as well as normal level of systolic and diastolic blood pressure.

None of the subjects was taking any systemic medications. In the POAG and PACG groups, patients were taking beta-blocker drops (33% and 46%, respectively), prostaglandins (38% and 23%), carbonic anhydrase inhibitor eye drops (21% and 21%) and alpha agonists (4% and 31%). In GODA group, patients were taking prostaglandins (21%), carbonic anhydrase inhibitor eye drops (6%) and alpha agonists (5%).

### Experimental Setup

Investigation of the ocular pulse characterized as the corneal indentation pulse (CIP) requires accurate instrumentation. This was first time pointed out by Hørven et al [28]. To register the CIP noninvasively and in a non-contact manner, we introduced an innovative ultrasonic system developed by UltraLab, Wroclaw, Poland. This system is based on an ultrasonic distance sensor functioning at a frequency of 0.8 MHz [43]. The transducer is placed in front of the cornea within its working distance (around 12 to 15 mm) and allows measuring the CIP amplitude with the accuracy below 1 µm [43]. The presented ultrasonic technique was utilized in previous *in vivo* ocular surface expansion studies [42]. Also, it was fundamental in establishing the presence of ocular dicrotic pulse in elderly subjects [29].

Simultaneously with the CIP, blood pulsation (BPL) and electrocardiogram (ECG) signals were recorded using pulseoximeter placed on the right earlobe of a subject and a standard three-lead system (Einthoven’s triangle) [44], respectively. BPL signal reflects oxygen saturation in the blood during each heartbeat, whereas the ECG signals the electrical heart activity in the form of P wave, QRS complex and T wave deflections describing atrial depolarization, depolarization and repolarization of the ventricles, respectively [44]. The sampling frequency for CIP, BPL and ECG signals was set to 400 Hz. The measurement time was 10 s, whereas a subject was instructed to fixate on a stationary target, set at their far point, and to keep their eyes open. Since, the panel of the ultrasonic system contains a preview of the received ultrasound echo signal in real-time, it has the possibility of monitoring potential blinks and considerable eye movements. For further analysis, the first measurement containing no blinks and no eye movements was saved.

During measurements, each subject was sitting in a relaxed position and breathing naturally [45]. Previous studies showed that a rigid headrest is required to minimize head movements during the ocular pulse measurement [42], [46]. For this purpose, we used a heavy headrest with an extra belt to strap a head of the subject to the headrest frame.

### Data Analysis

Saved data of CIP, BPL and ECG signals were numerically processed in a custom program written in Matlab (MathWorks, Inc. Natick, MA). Signal preprocessing included subtracting a linear trend and band-pass filtering from 0.5 Hz to 20 Hz. The filtering range was chosen to remove low frequencies (below 0.5 Hz) related to breathing modulation and to broaden the QRS complex of ECG signal.

Because of nonstationary nature of considered signals [25], averaging of their shapes is not obvious. In such signals, the time warping problem appears, meaning the local differences in the lengths of individual cardiac cycles of the signal occur. It is caused, mainly, by heart rate variability and respiratory sinus arrhythmia [47]. As it was presented in our recent work [29], to resolve the time warping problem of CIP, BPL and ECG signals, as well as to average their shapes the algorithm of Dynamic Time Warping (DTW) [48], [49] was utilized. The DTW procedure of averaging the considered signals from their whole records to one cardiac cycle (in the R to R range in ECG signal) was described in details in previous study [29]. This procedure was performed for all measurements of the current study.

To declare the ocular dicrotic pulse (ODP) corresponding to a double peak waveform of the CIP signal, a −3 dB criterion was used, based on the relative relationship between the peaks in the averaged signal waveform. In other words, when the valley between the two peaks in the averaged CIP signal was below a level corresponding to 0.707 of the lower of the two peaks an ODP type waveform was affirmed.

**Figure 1:**
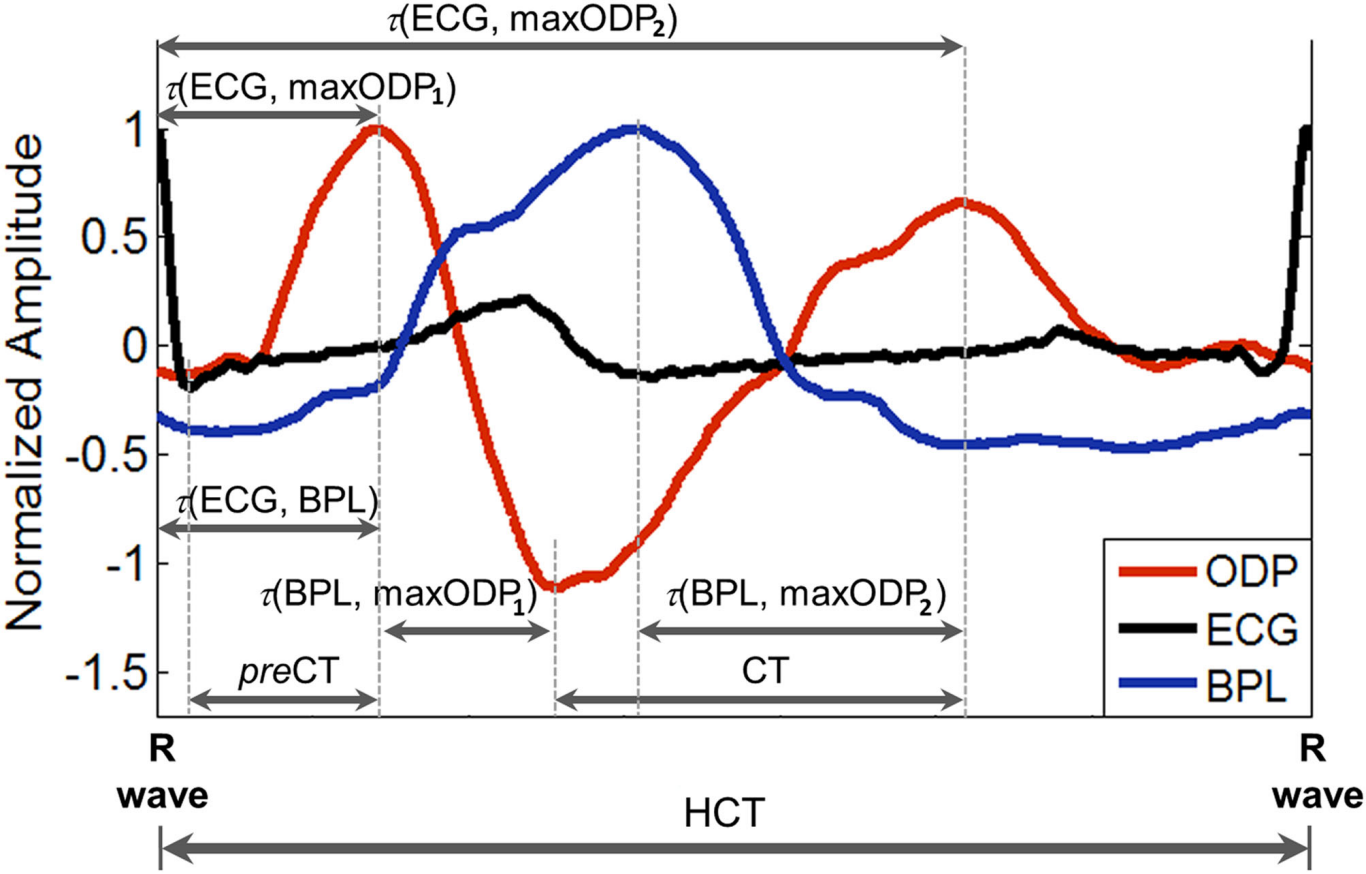
Illustration of typical averaged shapes of ODP, BPL and ECG signals for patient with PACG. The shapes of presented signals were averaged for one heart cycle and the considered signal parameters are following: *τ*(ECG, BPL) – time delay between R wave peak of ECG and systolic BPL peak; *τ*(ECG, maxODP_1_) – time delay between R wave peak of ECG and the first major ODP maximum; *τ*(ECG, maxODP_2_) – time delay between R wave peak of ECG and the second major ODP maximum; *τ*(BPL, maxODP_1_) – time delay between systolic BPL peak and the first major ODP maximum; *τ*(BPL, maxODP_2_) – time delay between systolic BPL peak and the second major ODP maximum; *pre*CT – preliminary crest time - time taken from minimum to the first major maximum of ODP shape; CT – crest time - time taken from minimum to maximum of ODP shape; HCT – time duration of heart cycle, taken from R peak to the next R peak of ECG shape.

Typical averaged ODP, BPL and ECG signals for one heart cycle for a PACG patient are illustrated in Figure 1.

The shape parameters of ODP waveform and the time delays between the first and second maximum of ODP signal, BPL peak and R wave of ECG signal were computed (see Figure 1):

- *τ*(ECG, BPL) - time delay between R wave peak of ECG and systolic BPL peak,
- *τ*(ECG, maxODP_1_) - time delay between R wave peak of ECG and the first major ODP maximum,
- *τ*(ECG, maxODP_2_) - time delay between R wave peak of ECG and the second major ODP maximum,
- *τ*(BPL, maxODP_1_) - time delay between systolic BPL peak and the first major ODP maximum,
- *τ*(BPL, maxODP_2_) - time delay between systolic BPL peak and the second major ODP maximum,
- *pre*CT - preliminary crest time - time taken from minimum to the first major maximum of ODP shape,
- CT - crest time - time taken from minimum to maximum of ODP shape,
- HCT - time duration of heart cycle, taken from R peak to the next R peak of ECG shape,
- *pre*RCT = *pre*CT/HCT*100% - preliminary relative crest time, and
- RCT = CT/HCT*100% - relative crest time.

Additionally, the heart rate (HR) of each examined subject was calculated based on their ECG records.

All subjects who exhibited the ocular dicrotism were divided into two age subgroups: less than or equal 63 years old (from 47 to 63 y.o.) and more than 63 years old (from 64 to 78 y.o., see Table 2). The age of 63 years was chosen as the median value of subjects’ age. These two age subgroups were taken to further analysis, within which all mentioned signal parameters were computed.

**Table 2:** Incidence of ODP in the two age subgroups.

	47÷63 y.o.			64÷78 y.o.			
	ODP [%]	Mean age (years±SD)	Number of subjects	ODP [%]	Mean age (years±SD)	Number of subjects	ODP incidence difference Δ [%]
**CG**	73	58.5±3.0	30	83	66.2±2.0	24	10
**GODA**	66	54.2±5.3	14	66	73.2±5.3	25	0
**POAG**	59	56.5±5.4	32	88	67.6±5.7	14	29
**PACG**	61	54.6±2.2	11	91	69.5±3.8	39	30
**ALL**	65	57.9±5.0	87	80	67.4±3.0	102	15

ODP – ocular dicrotic pulse; CG – control group; GODA – glaucoma suspects with glaucomatous optic disc appearance; POAG – primary open angle glaucoma; PACG – primary angle closure glaucoma.

### Statistics

Statistical analysis included standard descriptive statistics, hypothesis testing using the general linear model (two-way ANOVA, IBM SPSS Statistics ver. 21) that includes the Levene’s test for the equality of variances. The significance level of α = 0.05 was set for all considered tests. Results of the general linear model test were considered valid only for the cases where the hypothesis of the equality of variances was not rejected.

## Results

### Incidence of ODP

The incidence of ODP in all study participants and in each considered group of subjects together with their mean age and gender ratio, GAT IOP, CCT, mean defect (MD), Cup/Disk ratio, and systolic and diastolic blood pressure is shown in Table 1.

Overall, ODP was observed in 189 out of 261 subjects. In particular subject groups (CG, GODA, POAG and PACG) the ODP incidence was 77%, 65%, 65% and 82%, respectively.

To ascertain whether the incidence of ocular dicrotism increases with age, it was computed separately for two age subgroups (less than or equal to and more than the median age value of 63 years). The results are collected in Table 2.

For all subjects, the incidence of ocular dicrotism increases with age (Δ = 15%). For CG, POAG and PACG group the ODP incidence was higher in the older group of subjects (Δ = 10%, Δ = 29% and Δ = 30%, respectively). For GODA, however, no change in OPD incidence was observed (Δ = 0%).

### Signal parameter analysis

A multicomparison of means using the general linear model (two-way ANOVA) for the ten considered signal parameters: *τ*(ECG, BPL), *τ*(ECG, maxCIP_1_), *τ*(ECG, maxCIP_2_), *τ*(BPL, maxCIP_1_), *τ*(BPL, maxCIP_2_), *pre*CT, CT, *pre*RCT, RCT and HR was applied in order to provide the answer to the third question posed in the introduction. The results are shown in Table 3.

**Table 3:** The results of two way ANOVA (with the given signal parameter being the dependent variable and age and the subject group being the two independent variables).

		47÷63 y.o.	64÷78 y.o.	*p*-value			
No.	Parameter	Mean±SD	Mean±SD	Levene’s test	Age Group (≤63 &>63 y.o.)	Subject Group (CG,GODA, POAG, PACG)	Interaction SubjectGroup & Age
1	*τ* **(ECG, BPL) [s]**	0.28±0.03	0.31±0.07	0.02	-	-	-
2	*τ* **(ECG, maxCIP1) [s]**	0.16±0.04	0.18±0.05	0.41	**<0.001**	0.20	0.41
3	*τ* **(ECG, maxCIP2) [s]**	0.57±0.12	0.64±0.14	0.46	**0.003**	0.39	0.62
4	*τ* **(BPL, maxCIP1) [s]**	0.12±0.05	0.13±0.07	0.03	-	-	-
5	*τ* **(BPL, maxCIP2) [s]**	0.29±0.12	0.33±0.12	0.16	0.06	0.05	0.68
6	***pre*** **CT [s]**	0.12±0.03	0.13±0.04	0.34	**0.001**	0.08	0.31
7	**CT [s]**	0.25±0.09	0.30±0.11	0.22	0.06	0.05	0.91
8	***pre*** **RCT [%]**	16±5	16±4	0.04	-	-	-
9	**RCT [%]**	33±11	34±12	0.07	0.96	0.13	0.68
10	**HR [beat/min]**	79±10	71±11	0.14	**<0.001**	0.40	**0.01**

Statistically significant differences satisfying the criterion of equality of variances are marked in bold font.

CG – control group; GODA – glaucoma suspects with glaucomatous optic disc appearance; POAG – primary open angle glaucoma; PACG – primary angle closure glaucoma; *τ*(ECG, BPL) – time delay between R wave peak of ECG and systolic BPL peak; *τ*(ECG, maxODP_1_) – time delay between R wave peak of ECG and the first major ODP maximum; *τ*(ECG, maxODP_2_) – time delay between R wave peak of ECG and the second major ODP maximum; *τ*(BPL, maxODP_1_) – time delay between systolic BPL peak and the first major ODP maximum; *τ*(BPL, maxODP_2_) – time delay between systolic BPL peak and the second major ODP maximum; *pre*CT – preliminary crest time - time taken from minimum to the first major maximum of ODP shape; CT – crest time - time taken from minimum to maximum of ODP shape; HCT – time duration of heart cycle, taken from R peak to the next R peak of ECG shape; *pre*RCT = *pre*CT/HCT*100% – preliminary relative crest time; RCT = CT/HCT*100% – relative crest time and HR – the heart rate.

For age, four parameters: *τ*(ECG, maxCIP_1_), *τ*(ECG, maxCIP_2_), *pre*CT and HR were found to show statistically significant differences (*p*<0.05) between the two considered age subgroups. For the four groups of subjects (CG, POAG, PACG, GODA), there were no statistically significant differences in any of the ten signal parameters. Finally, a statistically significant interaction between the subject groups and age were found for HR.

## Discussion

Ocular dicrotic pulse (ODP) is a phenomenon, manifesting itself as a double-peak-shape of the corneal indentation pulse (CIP). Recently, it has been found to be present in the substantial proportion of older subjects [29]. The origin of ocular dicrotism is still unknown. However, the dicrotic arterial wave, also visible as the two distinct peaks, has been studied since 1873 [50]−[55]. It was shown to be linked to disturbances in arterial tension [46], [50], rigidity of arterial walls [54], as well as some cardiac failure and diseases [55]−[60].

In this study, we aimed at ascertaining whether the incidence of ODP in the elderly subjects is a function of age (see Q1 in the Introduction), it is more common in the glaucomatous eyes than in the normal ones (Q2) and whether it is related to cardiovascular activity (Q3).

The obtained results showed that ODP occurred in more than two-thirds of all examined subjects. This confirmed the results of our previous study [29], which showed 29 out of 41 older subjects to exhibit ocular dicrotism but was not focused on determining the incidence of ODP phenomenon in elderly group as a function of age.

With advancing age, the ODP incidence increased for all subjects. Worth noting is that the highest ODP occurrence (82%) and its age-related rise (Δ = 30%) were observed for the PACG group. It is the evidence that ODP is not only a natural sign of aging, but also may be an indication of progressing glaucoma or a consequence of medications taken by the subjects. Only for GODA, the ODP incidence remained unchanged with increasing age (Δ = 0%). This group also took medications but not the beta blockers. Studies of the effects of beta-blockers on ocular blood flow in glaucomatous eyes revealed that some of the have a vasodilator effect while other have no effect on the retinal and choroidal vasculature [61]. However, the effect of beta blocker drops and other medications on the characteristics of the CIP is yet to be determined and it outside the scope of this study.

Further, signal parameters such as: *τ*(ECG, maxCIP_1_), *τ*(ECG, maxCIP_2_), *pre*CT and HR, showed statistically significant differences in relation to age. Also, HR was found to be statistically significant in an interaction between age and subject groups. Beyond *pre*CT, there were three parameters closely corresponding to the heart activity, which indicate a substantial role that age-related changes in cardiac activity could play in the ODP phenomenon.

The *pre*CT was the only parameter of the ODP waveform that showed statistically significant dependence with age. It underlines the importance of the first peak in the ODP signal in the studies of human eye aging.

Age-related biomechanical changes in the eye were linked to higher ocular rigidity [13]. Effect of eye aging was observed also in other conditions, such as alterations in ocular blood flow [7], [8], especially choroidal circulation [9] or structural and biochemical changes of corneal stroma [2]−[5]. Furthermore, with advancing age, the tissues of the sclera and lamina cribrosa were found to become stiffer in human eyes [6]. Watson and colleagues [62] found that thickness of human sclera increases with age. It is plausible that the observed ODP signal is the result of combinations of the above mentioned factors.

Natural aging causes also cardiovascular changes. For example, it increases arterial stiffness and pulse wave velocity [63], [64] as well as changes the contour of arterial pulse wave in various body locations [65]−[69]. Due to a close relationship between the corneal pulsation and heart activity signals [25]−[27] it is very important to include the role of cardiovascular activity system in characterization of the ODP signal.

Hence, ocular dicrotism observed in our study can be interpreted as a mechanical corneal response to a propagation of an ocular blood pressure wave to the interior of the eye with each heartbeat that is closely related to age-related factors of ocular elasticity and hemodynamics.

Furthermore, aging is also considered a factor in development of many ocular diseases, such as glaucoma [30], [69]. Higher ocular rigidity was observed in glaucoma eyes than in the normal eyes [13], [70], [71]. Studies show increased eye stiffness of glaucoma patients pointing to changes in the structural design of the sclera [72], [73], or more specifically to the change in the composition of collagen fibers [74]. Various experimental techniques, including *in-vitro* [72] and *in-vivo* [70], [71], were applied to characterize eye tissues response in glaucoma and age-matched subjects.

Ebneter *et al*. [71] considered indirectly assessing ocular rigidity by measuring IOP and axial eye length in groups of POAG and the controls. The comparison of those two groups showed that patients with established glaucoma (POAG) presented an increased ocular rigidity. Also, a coefficient of ocular rigidity, calculated from IOP and fundus pulsation amplitudes measured with pneumotonometry and laser interferometry, respectively, was significantly higher in POAG group [70].

Biomechanical responses of eye tissues have been tested in numerous animal models with exposure to chronic IOP elevations [75]–[78]. McDowell *et al*. [76] demonstrated mouse model of human POAG and strain differences in induced ocular hypertension and pressure-induced optic nerve damage. Girard *et al*. [77] suggested greater stiffness in monkey glaucoma eyes at moderate stages of glaucoma. The animal experimental models suggest that greater stiffness is an effect of glaucoma. Hence, different biomechanical behaviors of cornea and sclera to chronically elevated IOP, such as the measured in our study increase in the incidence of ODP in both glaucoma groups, may provide important clues to the risk indicators in explaining pathogenesis of this disease.

Additionally, reduced fundus pulsation amplitude and pulsatile ocular blood flow (POBF) were found in patients with POAG compared to the age-matched control subjects [70]. This confirms previous studies concerning decreased POBF in POAG group [79]−[83] and highlights vascular abnormalities as a factor of POAG progression and incidence.

In relation to the POAG, GODA and normal groups, impaired optic nerve blood flow was detected in POAG, as well as in GODA subjects [80]. However, spontaneous venous pulsation was observed to be less common phenomenon in POAG patients than in glaucoma suspects [84]. Alteration in ocular hemodynamics in POAG and normal tension glaucoma groups has been extensively investigated [69], . However, its dysregulative mechanism is still unclear [91].

There is less data considering ocular rigidity and ocular blood flow parameters in PACG subjects. Our results indicate that ODP incidence in the PACG group is similar to that of the POAG. Expansion of choroid was demonstrated as a risk factor contributing mechanism to development of PACG [92], [93]. Based on those findings, we speculate that the increased ODP incidence found in PACG and POAG group might reflect higher stiffness of glaucoma eyes and in some extent correspond to changes in the intraocular vascular bed and biochemical abnormalities in eye tissues.

Of particular interest is the result for the GODA group which did not show an age-related increase in the incidence of ODP despite the group having the greatest, among the four considered subject groups, average age difference between the two subgroups (i.e., less than or equal to 63 y.o. and more than 63 y.o). One could speculate that in GODA subjects the age-related changes in ocular elasticity may not necessarily positively correlate with factors related to the impaired optic nerve blood flow or other abnormalities in eye biochemistry making them less susceptible to generating ODP. To date there is not enough experimental evidence to substantiate this supposition. Nevertheless, synchronous measurement of ocular pulse and ocular blood flow [94] as well as the electrical heart activity signals may, in the future, point to those aspects of aging that are responsible for the increased predisposition to glaucoma.

## Supporting Information

Raw Data S1 A zipped file containing three databases of CIP parameters for the group of subjects with no dicrotism and for subjects with ODP younger than or equal to the median age of 63 years and for those older than the median age.
(RAR)
